# Abdominal actinomycosis mimicking a transverse colon malignancy: a case report and review of the literature

**DOI:** 10.1186/s13256-021-02812-7

**Published:** 2021-05-03

**Authors:** Gnanaselvam Pamathy, Umesh Jayarajah, Dayal Sathyajith Gamlaksha, Roshana Constantine, Anura S. K. Banagala

**Affiliations:** 1 Department of Surgery, National Hospital of Sri Lanka, Colombo 10, Sri Lanka; 2 Department of Pathology, National Hospital of Sri Lanka, Colombo, Sri Lanka

**Keywords:** Actinomycosis, Intra-uterine device, Transverse colon, Case report

## Abstract

**Background:**

Actinomycosis is a rare inflammatory bacterial disease caused by *Actinomyces* species which can infrequently affect the large intestine. Disseminated actinomycosis is reported as a rare complication associated with intrauterine devices. We report a case of intra-abdominal actinomycosis mimicking a transverse colon malignancy.

**Case presentation:**

A previously healthy 40-year-old Sinhalese woman was evaluated for intermittent colicky left-sided abdominal pain for 2 months’ duration. Computed tomography of the abdomen showed a circumferential thickening of the wall and narrowing of the lumen of the descending colon with evidence of extraluminal extension to the adjacent parietal peritoneum and abdominal wall suggestive of a stage IV neoplasm. An exploratory laparotomy with extended left hemicolectomy was performed. Macroscopic evaluation revealed a mass lesion with multiple abscesses attached to the transverse and descending colon. Histology was suggestive of actinomycosis with no evidence of malignancy.

**Conclusions:**

Abdominal actinomycosis should be considered in a young patient with chronic abdominal pain. It should be understood that the presentation may be vague and highly variable. Computed tomography-guided biopsy/fine needle aspiration or laparoscopy and biopsy may be useful in arriving at a diagnosis and can prevent unnecessary surgical intervention.

## Background

Actinomycosis is a rare inflammatory condition caused by *Actinomyces* species, which are anaerobic bacteria [1]. They are gram-positive bacteria, which are part of the normal human flora. *Actinomyces* requires the presence of an anaerobic microenvironment to proliferate, and therefore requires the concomitant presence of many other bacteria for this purpose [1]. Actinomycosis associated with the presence of intrauterine devices is reported in the literature and it mainly involves the pelvis [2]. Actinomycosis primarily involving the bowel is rare, and the most common sites are the terminal ileum and the cecum [3, 4].

Clinical and radiological features of actinomycosis can mimic other benign and malignant abdominal diseases such as tuberculosis, diverticulitis, inflammatory bowel disease and malignancies. Therefore, the diagnosis is usually challenging and the disease is often detected histologically after surgery [5].

In this case report, we present a rare occurrence of intra-abdominal actinomycosis mimicking a large intestinal tumor.

## Case presentation

A 40-year-old Sinhalese woman who was previous healthy presented with a history of intermittent colicky left-sided abdominal pain for 2 months’ duration. There were no other lower gastrointestinal symptoms, loss of weight or loss of appetite. She was hemodynamically stable. There was no palpable abdominal mass or evidence of peritonitis, and the rest of the history and examination were unremarkable. She did not have a significant family history of illnesses. She was unemployed. Basic biochemistry, which included complete blood count and renal and liver profile, was within normal parameters. Colonoscopy revealed a narrowing of the lumen of the descending colon a few centimeters distal to the splenic flexure. Biopsy showed evidence of resolving infective colitis, with no evidence of malignancy. Computed tomography of the abdomen and colonogram showed a circumferential thickening of the wall and narrowing of the lumen of the descending colon extending from 3 cm below the splenic flexure downwards, with evidence of extraluminal spread through the serosa and infiltration of adjacent parietal peritoneum and abdominal wall, suggestive of a stage IV neoplasm. There was no evidence of abdominal metastasis.

Surgical exploration and excision of a probable malignancy was planned. Exploratory laparotomy revealed a mass lesion with multiple abscesses attached to the transverse and descending colon with a narrowed segment at the proximal descending colon. The mass was attached to the omentum and pericolic fat. Multiple enlarged lymph nodes along the inferior mesenteric artery were noted. The patient underwent extended left hemicolectomy with end-to-end colocolic anastomosis.

The surgical specimen consisted of transverse colon and descending colon together measuring 370 mm in length and 50 mm in diameter. The lumen of the descending colon was narrowed in a region 130 mm from the proximal resection margin. A mass lesion with multiple abscesses measuring 90 x 70 x 45 mm was attached to the transverse and descending colon at the narrowed segment.

**Fig. 1:**
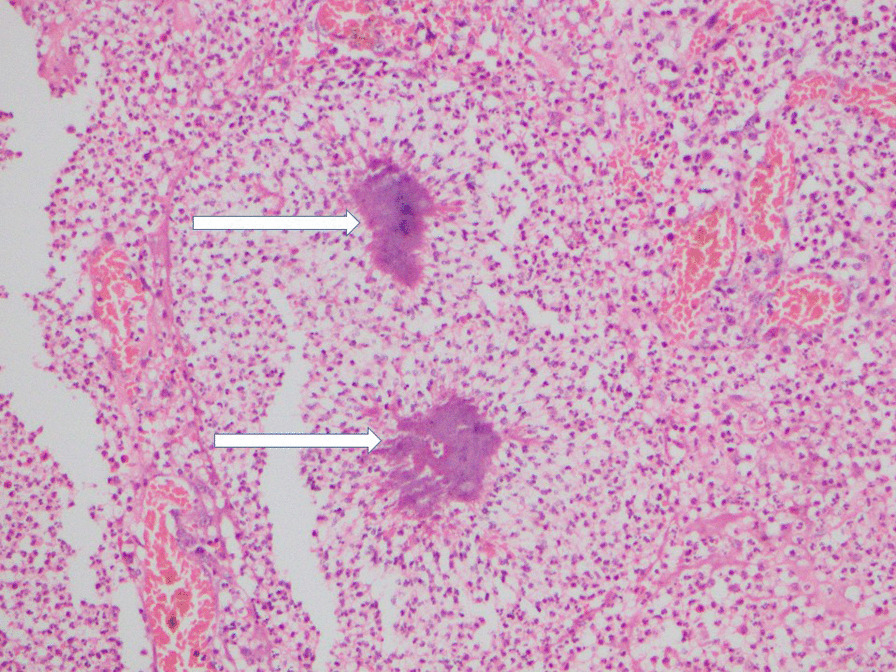
Section of intestinal wall: *Actinomyces* colony (white arrows) showing basophilic radiating filaments. Hematoxylin and eosin, ×100

**Fig. 2:**
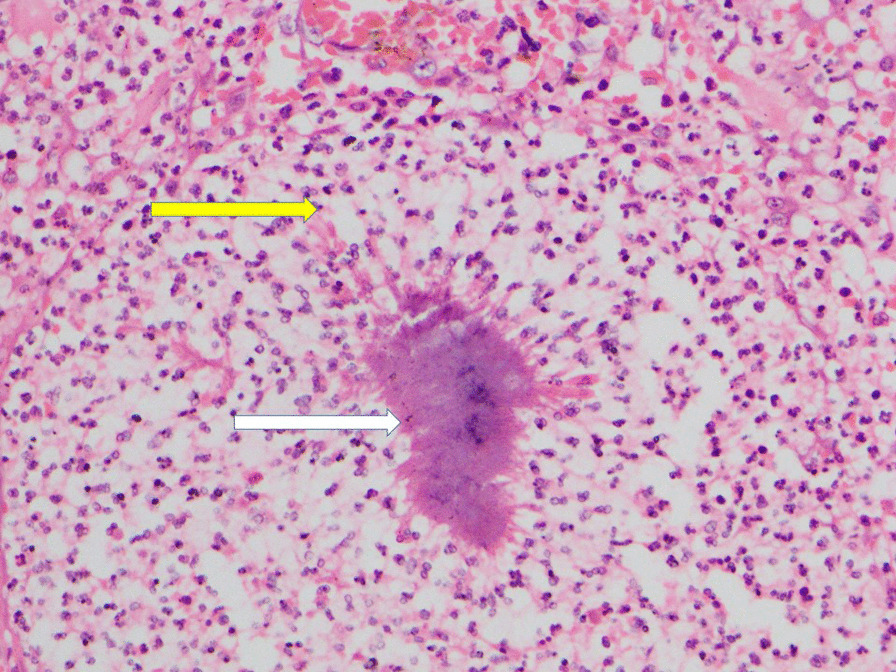
Section of intestinal wall: *Actinomyces* colony (white arrow) surrounded by neutrophil-predominant inflammatory cells (yellow arrow). Hematoxylin and eosin, ×200

Microscopic examination of sections of the mass lesion showed multiple abscesses surrounded by a fibroblastic reaction and a mixed inflammatory infiltrate with foreign body type giant cells. Occasional abscesses contained a few basophilic colonies suggestive of actinomycosis. The lymph nodes showed reactive changes. There was no evidence of tuberculosis, inflammatory bowel disease or malignancy (Figs. 1, 2).

The patient received intravenous penicillin treatment for 10 days followed by oral penicillin for 6 months after reviewing with histology. There were no complications related to surgery. Follow-up assessment after 1 year revealed that the patient was free of symptoms, with no clinical evidence of bowel obstruction.

## Discussion

*Actinomyces israelii* is a filamentous gram-positive bacillus which is a commonly seen constituent of the microflora in the human body [6]. Actinomycosis has been reported in many parts of the world. Though most reported cases of actinomycosis were in males, a preponderance to a specific sex is not proven.

Involvement of the abdominal organs was reported in up to 20% of all cases of actinomycosis. The clinical presentation may vary and is known to mimic malignant tumors, inflammatory bowel disease and tuberculosis [7].

Actinomyces is a part of the normal flora in the gut and female genital tract. In the gut, it is normally found in the stagnated segments which are the cecum and sigmoid colon. It normally does not cause any disease. However, factors such as intestinal necrosis, previous abdominal surgeries, foreign bodies, appendicitis and perforation can predispose to infections. The exact mechanism is not known, and some authors postulate that neoplastic or inflammatory processes may contribute to actinomycosis development [8, 9]. Similarly, we could not identify a cause in our patient.

Actinomycosis is commonly known to occur in the terminal ileum, cecum and appendix and less commonly in the ascending colon. Obstruction secondary to actinomycosis is rare, as the typically affected region of the bowel contains liquid material and has a wider lumen. The involvement of the transverse and the descending colon is rare, which is the region of involvement in the reported case.

Actinomycosis has a vast spectrum of presentations. It is known to present with bowel obstruction [10], multiple abscesses and draining sinuses [11], bowel masses mimicking malignancies [1, 12, 13], bowel perforation [3, 14] and acute appendicitis [15]. Sometimes patients present with nonspecific symptoms such as abdominal pain, fever and weight loss similar to the reported case [16]. Due to the rarity of actinomycosis, other sinister causes are given priority, which leads to unnecessary morbidity related to delayed diagnosis and surgery. However, the indolent, slowly progressive nature of the condition is an important clue to the diagnosis, as in this case.

Often, actinomycosis is diagnosed postoperatively, although in certain instances histopathological assessment of endoscopically obtained specimens may help in the diagnosis. When actinomycosis presents as bowel obstruction or abdominal mass mimicking a malignancy, the diagnosis by imaging becomes difficult. However, contrast-enhanced computed tomography (CT) scan may sometimes reveal an intra- or extraluminal solid mass with focal areas of attenuation invading the nearby tissues [17, 18]. Often, the radiological findings are similar to those of malignancies, abdominal tuberculosis or inflammatory bowel disease. The commonest findings in CT scan or barium study are mural invasion with formation of stricture, tapered narrowing of the lumen secondary to a mass effect, and thickened mucosal folds. However, these features are also seen in the above conditions and thus are not specific to actinomycosis [19].

Furthermore, a common finding includes a large mass adjacent to the involved bowel, which is seen in actinomycosis of the colon. In rectosigmoid regions, cystic masses are more commonly seen, while in transverse or ascending colon regions, pure solid masses are commonly noted. These features may help in arriving at a diagnosis [20]. In later stages of the disease, chronic inflammation leading to infiltration across tissue planes may occur, with formation of multiple fistula tracts to the abdominal wall, perineum, other nearby organs or between bowel loops [18].

Histopathological and microbiological analyses are key to the diagnosis. A definitive diagnosis is achieved by the presence of sulfur granules and/or culture of *Actinomyces* obtained from a surgical specimen or needle aspiration of a collection. Endoscopic biopsies usually result in a limited study and only demonstrate chronic inflammation without sulfur granules. This is because of the limited depth of tissue sampling. Sulfur granules represent colonies of *Actinomyces.* Although this is highly suggestive, it is not a pathognomonic feature of actinomycosis. Sulphur granules may be seen in nocardiosis, botryomycosis and *Aspergillus* infections [16]. Therefore, CT-guided fine needle aspiration (FNA) may be helpful in diagnosis. However, in the majority of cases, obtaining a proper sample is difficult and challenging when the small or large bowel is involved. Furthermore, the samples may not adequately represent the area of active disease, resulting in false-negative histology [11]. In retrospect, a CT-guided biopsy may have been helpful in our patient to obtain a better histological diagnosis. However, due to the concern of a possible malignancy and the questionable yield of guided biopsy, we opted for surgery.

High-dose intravenous penicillin administration followed by prolonged oral penicillin treatment for at least 6–12 months is the treatment of choice. Penicillin is known to decrease morbidity and unnecessary invasive procedures such as laparotomy [21]. However, in the majority of cases, the diagnosis is reached postoperatively (90%) but it is still mandatory to administer the complete course of penicillin [1]. The total duration of antibiotics depends on the initial nature and extent of disease and the clinical and radiological response to treatment. Surgery is offered selectively for large abscesses, bulky necrotic lesions, fistulas or lesions with suspicion of a malignancy. The prognosis is favorable in more than 90% of cases after medical and surgical therapy [15].

## Conclusions

In conclusion, colonic actinomycosis should be considered in a young patient presenting with chronic abdominal pain. It should be understood that the presentation may be highly variable, with vague symptoms. Therefore, a high degree of clinical suspicion is essential for diagnosis. CT-guided biopsy/FNA or laparoscopy and biopsy may be useful in arriving at a diagnosis that can prevent unnecessary invasive surgical interventions.

## Data Availability

All data generated and/or analyzed during this study are included in this published article.

## Abbreviations

CT: Computed tomography
FNA: Fine needle aspiration
